# Development and validation of machine learning-based prediction for in-hospital mortality in ICU patients with severe community-acquired pneumonia and respiratory failure

**DOI:** 10.3389/fmed.2026.1743986

**Published:** 2026-04-20

**Authors:** Xing Zheng, Bingxian Wang, Li Yuan, Xiaoqin Liu, Ying Xu, Bin Sun

**Affiliations:** 1 Department of Intensive Care Unit, National Regional Medical Center for Qinghai (Qinghai Hospital), The First Affiliated Hospital of Xi'an Jiaotong University, Xining, Qinghai, China; 2 Department of Emergency Intensive Care Unit, Qinghai Provincial People's Hospital, Xining, Qinghai, China; 3 Department of Anesthesiology, Qinghai Provincial People's Hospital, Xining, China; 4 Department of Intensive Care Unit, Qinghai Provincial People's Hospital, Xining, Qinghai, China

**Keywords:** intensive care unit, machine learning, mortality risk prediction, respiratory failure, severe community-acquired pneumonia

## Abstract

**Background:**

Accurate prediction of in-hospital mortality for patients with severe community-acquired pneumonia (SCAP) complicated by respiratory failure admitted to the intensive care unit (ICU) remains a critical challenge. This study aimed to develop and validate a machine learning (ML) model to predict this risk and compare its performance with conventional scoring systems.

**Methods:**

In this retrospective study, data from 164 patients with SCAP and respiratory failure admitted to the ICU between January 2017 and January 2024 were analyzed. Patients were randomly divided into a training set (*n* = 114) and a validation (test) set (*n* = 50). Forty-five clinical features collected at admission were used as candidate predictors. The Least Absolute Shrinkage and Selection Operator (LASSO) regression was employed for feature selection. Six ML models, including Gradient Boosting Decision Tree (GBDT), Random Forest (RF), eXtreme Gradient Boosting (XGBoost), Decision Tree(DT),Support Vector Machine (SVM), and Logistic Regression(LR), were constructed and evaluated.Use SHAP analysis to assess the contribution of each feature in a machine learning model. Construct a nomogram using the top six most influential features.

**Results:**

The GBDT model demonstrated the best predictive performance, achieving an area under the receiver operating characteristic curve (AUC) of 0.83 (95% CI: 0.757–0.927) in the internal validation set, significantly outperforming the Acute Physiology and Chronic Health Evaluation II (APACHE-II, AUC = 0.70). Calibration curves demonstrated good agreement between predicted and observed mortality risks, particularly across the mid-probability range. Decision curve analysis indicated that the model provided a higher net benefit than “treat-all” and “treat-none” strategies across a broad range of threshold probabilities. SHapley Additive exPlanations (SHAP) analysis identified lactate, D-dimer, temperature, albumin, Prothrombin Time and Fraction of Inspired Oxygen as the six most influential predictors of in-hospital mortality. Based on these key predictors, we further developed a simplified nomogram to facilitate bedside risk estimation.

**Conclusion:**

The GBDT ML model, developed from routinely available clinical data, provides a highly accurate and clinically interpretable tool for predicting in-hospital mortality in SCAP patients with respiratory failure. It outperforms traditional severity scores and holds promise for assisting clinicians in risk stratification and early intervention.

## 1 Introduction

Severe community-acquired pneumonia (SCAP) remains a leading cause of critical illness and mortality worldwide, often progressing to acute respiratory failure and necessitating admission to the Intensive Care Unit (ICU) (1–3). The in-hospital mortality for this specific patient population is notoriously high, placing a significant burden on healthcare systems (4). The timely and accurate prediction of mortality risk is not merely a prognostic exercise but a clinical imperative, as it facilitates the early identification of high-risk patients, informs therapeutic strategy intensification, and improves the allocation of critical care resources.

In current clinical practice, the prognosis of critically ill patients is commonly assessed using generalized severity-of-illness scores, such as the Acute Physiology and Chronic Health Evaluation II (APACHE II) (5–8). While these scoring systems provide a valuable snapshot of a patient's physiological derangement, they possess inherent limitations when applied to specific patient cohorts like those with SCAP and respiratory failure (6, 9, 10). Their predictive accuracy is often suboptimal, as they may not fully capture the complex, non-linear interactions between the myriad of clinical variables unique to this disease process. Consequently, there is a pressing need for more tailored and accurate predictive tools.

The advent of machine learning (ML) in healthcare offers a promising avenue to address this challenge (11). Unlike traditional statistical models that often rely on pre-specified linear relationships, ML algorithms can automatically learn complex patterns from high-dimensional clinical data (11–14). Techniques such as Gradient Boosting Decision Trees (GBDT) have demonstrated superior performance in various medical prediction tasks by effectively modeling intricate interactions between predictors (15–20). Furthermore, the development of model interpretation tools, such as SHapley Additive exPlanations (SHAP), has mitigated the “black-box” concern often associated with ML models, allowing clinicians to understand the contribution of each feature to the individual prediction (21–25).

Therefore, the primary objective of this study was to develop and validate a robust ML-based model specifically designed to predict in-hospital mortality for ICU patients with SCAP and respiratory failure. We systematically compared the performance of multiple ML algorithms against each other and against traditional scoring systems. We hypothesize that a tailored ML model will significantly outperform conventional APACHE II in predictive accuracy, calibration, and clinical utility, ultimately providing a more reliable tool for risk stratification in this vulnerable population.

## 2 Methods

### 2.1 Study design and data collection

This retrospective observational study was conducted in strict accordance with the principles of the Declaration of Helsinki. The study protocol received approval from the Ethics Committee of Qinghai Provincial People's Hospital. Given the retrospective nature of the research, the requirement for informed consent was waived. The study consecutively enrolled adult patients with severe pneumonia combined with respiratory failure who were admitted to the Intensive Care Unit (ICU) of our hospital between January 2017 and January 2024. Inclusion criteria were: (1) age ≥ 18 years; and (2) ICU admission with a primary diagnosis of severe pneumonia and respiratory failure. Exclusion criteria comprised: (1) pre-existing pulmonary diseases or pulmonary malignancies; (2) incomplete clinical data; or (3) transfer out of the ICU within 72 hours of admission.

### 2.2 Data collection and outcome definition

Baseline patient information was collected, including demographic data (sex, age) and comorbidities (diabetes, hypertension, coronary heart disease). Disease severity upon admission was assessed using the Acute Physiology and Chronic Health Evaluation II (APACHE II) score score. Vital signs and a range of laboratory parameters were also recorded. The primary outcome measure for this study was defined as all-cause in-hospital mortality. Based on this outcome, patients were categorized into survivor and non-survivor groups for comparative analysis.

### 2.3 Prediction model development

Initially, 45 clinical features readily available in routine clinical practice were included as candidate predictor variables. All eligible study subjects were randomly divided into a training set and a validation (test) set in a 7:3 ratio.

We employed Least Absolute Shrinkage and Selection Operator (LASSO) regression for the initial feature selection to enhance model performance and mitigate multicollinearity. The optimal regularization parameter (λ) was determined by 10-fold cross-validation, balancing the trade-off between feature sparsity and model accuracy.

Subsequently, six distinct machine learning algorithms were employed to construct prognostic prediction models based on the important variables identified by the LASSO regression. These algorithms included: Gradient Boosting Decision Tree (GBDT), Random Forest (RF), eXtreme Gradient Boosting (XGBoost), Logistic Regression, Support Vector Machines (SVM), and Decision Tree (DT).

### 2.4 Model validation and evaluation

The discriminative ability of each model was assessed using the area under the receiver operating characteristic curve (AUROC) on the independent validation set, and the best-performing machine learning model was further compared with the conventional APACHE-II score. Model calibration was evaluated by visual inspection of calibration curves and quantitatively using the Brier score; uncertainty of calibration performance was estimated via bootstrap resampling (200 resamples) in the validation cohort. Clinical usefulness was examined using decision curve analysis (DCA). DCA was interpreted in the context of an early ICU risk-stratification decision—whether to trigger an enhanced high-risk management pathway (e.g., closer monitoring, more frequent reassessment, and early senior/multidisciplinary review) based on predicted mortality risk—rather than to mandate a single treatment. Threshold probability (ptp_tpt) was defined as the risk level at which initiating this pathway would be justified given the trade-off between false-positive escalation and missed high-risk cases; because these actions are generally low-to-moderate cost and reversible in routine ICU practice, we considered pt≈0.10–0.60p_t \approx 0.10–0.60pt≈0.10–0.60 as the most clinically actionable range while presenting DCA results across the full range of thresholds.

### 2.5 Model interpretability analysis

The SHapley Additive exPlanations (SHAP) framework was applied to interpret the best-performing model. This approach quantifies the contribution of each feature to the model's predictions, providing a clear and quantitative representation of the direction and magnitude of each variable's influence on the outcome.

### 2.6 Nomogram construction

To enhance clinical usability, we constructed a nomogram to translate the most influential model predictors into an intuitive bedside tool. First, we performed multivariable logistic regression on the training set including the ten most salient predictors identified by the LASSO/SHAP framework to examine independent associations. For practical bedside risk estimation, we further developed a simplified nomogram incorporating the six highest-ranking SHAP features [lactate (Lac), D-dimer (DD), Temperature, albumin (ALB), Fraction of Inspired Oxygen, and tprothrombin time] using the “rms” package in R. Total points were calculated by summing variable-specific scores and mapped to an estimated probability of in-hospital mortality.

### 2.7 Statistical analysis

The normality of continuous variables was assessed using the Kolmogorov-Smirnov test. Normally distributed data are presented as mean ± standard deviation and were compared between groups using the Student's *t*-test. Non-normally distributed data are presented as median (interquartile range) and were compared using the Mann-Whitney *U*-test. Categorical variables are expressed as frequency (percentage) and were compared using the Pearson chi-square test. All statistical analyses were performed using Python (version 3.10.10). A two-sided *P*-value <0.05 was considered statistically significant.

## 3 Results

### 3.1 Baseline characteristics and dataset partition

A total of 164 patients with severe pneumonia combined with respiratory failure were ultimately included in this study and randomly allocated into a training set (*n* = 114) and a validation set (*n* = 50) at a 7:3 ratio. The mean age of patients in the training set was 63.10 ± 16.28 years, with males comprising 65.79% (75 cases) and 45 mortality cases (39.47%). In the validation set, the mean age was 60.64 ± 16.01 years, with males accounting for 76.00% (38 cases) and 22 mortality cases (44.00%). Comparative analysis of baseline characteristics between the two sets is presented in (Table 1). All variables, except for serum calcium (Ca^2^), showed no statistically significant differences (*P* > 0.05), indicating a well-balanced dataset partition (Table 1).

**Table 1:** Balance test between training set and validation set.

Variables	Total (*n* = 164)	Validation (*n* = 50)	Training (*n* = 114)	Statistic	*P*
Age, Mean ± SD	62.35 ± 16.19	60.64 ± 16.01	63.10 ± 16.28	t = −0.89	0.373
SIRS, Mean ± SD	2.31 ± 0.87	2.32 ± 0.84	2.31 ± 0.88	t = 0.09	0.930
APACHEII, M (Q1, Q3)	17.00 (13.00, 23.00)	17.00 (13.25, 22.75)	17.00 (13.00, 23.00)	Z = −0.01	0.990
SOFA, M (Q1, Q3)	6.00 (4.00, 9.00)	6.00 (4.25, 9.00)	6.00 (4.00, 8.00)	Z = −0.80	0.423
GCS, M (Q1, Q3)	15.00 (11.00, 15.00)	15.00 (11.25, 15.00)	15.00 (11.00, 15.00)	Z = −0.67	0.505
PH, M (Q1, Q3)	7.43 (7.33, 7.48)	7.44 (7.31, 7.48)	7.42 (7.34, 7.48)	Z = −0.07	0.947
Pa0_2_, M (Q1, Q3)	65.50 (55.00, 78.00)	59.50 (51.50, 76.25)	68.00 (55.92, 78.00)	Z = −1.52	0.128
FiO_2_, M (Q1, Q3)	0.60 (0.45, 0.81)	0.60 (0.40, 0.97)	0.60 (0.50, 0.80)	Z = −0.67	0.506
Lac, M (Q1, Q3)	1.90 (1.30, 3.10)	1.90 (1.40, 2.65)	1.85 (1.30, 3.10)	Z = −0.22	0.826
PCT, M (Q1, Q3)	1.17 (0.25, 5.01)	1.17 (0.28, 2.90)	1.12 (0.22, 5.71)	Z = −0.19	0.853
BNP, M (Q1, Q3)	382.73 (165.75, 867.25)	350.00 (163.50, 860.63)	385.10 (171.00, 858.21)	Z = −0.38	0.706
WBC, M (Q1, Q3)	10.79 (7.16, 15.29)	10.29 (5.72, 15.39)	10.92 (7.28, 15.13)	Z = −0.57	0.570
LY, M (Q1, Q3)	0.48 (0.30, 0.91)	0.56 (0.30, 0.94)	0.47 (0.30, 0.89)	Z = −0.37	0.714
HGB, M (Q1, Q3)	130.00 (110.75, 154.00)	133.00 (109.25, 150.00)	128.50 (112.00, 155.00)	Z = −0.41	0.680
PLT, M (Q1, Q3)	163.00 (104.25, 222.25)	161.50 (92.00, 217.25)	163.00 (111.50, 222.75)	Z = −0.50	0.614
TBIL, M (Q1, Q3)	19.10 (13.19, 27.55)	16.12 (12.65, 25.25)	20.09 (14.22, 27.67)	Z = −1.62	0.105
ALB, M (Q1, Q3)	26.95 (23.50, 29.90)	26.50 (23.13, 28.78)	27.45 (24.55, 30.58)	Z = −1.80	0.071
BUN, M (Q1, Q3)	7.96 (5.96, 11.61)	8.12 (6.23, 11.23)	7.75 (5.82, 11.68)	Z = −0.41	0.680
CREA, M (Q1, Q3)	71.50 (55.00, 98.50)	72.00 (50.25, 88.50)	71.00 (57.25, 100.75)	Z = −0.58	0.562
K, M (Q1, Q3)	3.88 (3.55, 4.23)	3.89 (3.55, 4.19)	3.87 (3.55, 4.23)	Z = −0.08	0.933
Na, M (Q1, Q3)	137.00 (133.75, 140.00)	137.00 (133.25, 139.00)	138.00 (134.00, 141.00)	Z = −0.79	0.428
Cl, M (Q1, Q3)	104.10 (100.38, 106.93)	103.85 (100.82, 107.22)	104.35 (99.35, 106.90)	Z = −0.07	0.944
Ca, M (Q1, Q3)	1.94 (1.82, 2.04)	1.90 (1.79, 2.00)	1.95 (1.84, 2.05)	Z = −1.99	**0.047**
P, M (Q1, Q3)	1.02 (0.78, 1.31)	1.04 (0.84, 1.30)	1.02 (0.77, 1.31)	Z = −0.29	0.770
Mg, M (Q1, Q3)	0.84 (0.76, 0.91)	0.83 (0.77, 0.91)	0.84 (0.75, 0.91)	Z = −0.20	0.840
CRP, M (Q1, Q3)	12.11 (6.54, 22.47)	12.57 (7.13, 23.19)	11.49 (6.45, 19.96)	Z = −0.25	0.805
GLU, M (Q1, Q3)	7.59 (6.04, 10.19)	7.57 (6.53, 10.09)	7.62 (6.04, 10.13)	Z = −0.28	0.779
PT, M (Q1, Q3)	14.15 (13.20, 16.33)	13.90 (13.25, 15.80)	14.25 (13.20, 16.38)	Z = −0.32	0.752
APTT, M (Q1, Q3)	33.30 (28.45, 41.52)	35.45 (28.90, 39.53)	33.20 (27.45, 43.15)	Z = −0.37	0.709
INR, M (Q1, Q3)	1.23 (1.12, 1.41)	1.21 (1.13, 1.38)	1.23 (1.11, 1.42)	Z = −0.36	0.720
FIB, M (Q1, Q3)	4.11 (3.17, 5.70)	3.96 (3.11, 5.87)	4.17 (3.18, 5.68)	Z = −0.16	0.869
DD, M (Q1, Q3)	5.94 (3.77, 12.54)	6.22 (3.81, 12.56)	5.86 (3.75, 12.26)	Z = −0.62	0.533
FDP, M (Q1, Q3)	15.77 (9.01, 27.68)	16.99 (10.76, 26.87)	15.43 (8.73, 27.87)	Z = −0.88	0.379
Body temperature, M (Q1, Q3)	36.80 (36.50, 37.23)	37.00 (36.50, 37.20)	36.80 (36.50, 37.30)	Z = −0.08	0.936
Pulse, M (Q1, Q3)	110.50 (97.00, 126.25)	114.50 (96.50, 127.75)	110.00 (97.00, 125.75)	Z = −0.53	0.598
Breathe, M (Q1, Q3)	29.00 (23.75, 35.00)	30.00 (25.00, 35.00)	28.00 (23.00, 35.00)	Z = −1.07	0.284
SBP, M (Q1, Q3)	126.00 (110.00, 144.25)	122.50 (102.25, 138.75)	129.00 (111.00, 148.75)	Z = −1.79	0.073
DBP, M (Q1, Q3)	80.00 (67.75, 89.00)	80.50 (67.25, 91.50)	79.00 (69.00, 88.00)	Z = −0.43	0.669
Gender, *n* (%)				χ^2^ = 1.69	0.193
1	113 (68.90)	38 (76.00)	75 (65.79)		
2	51 (31.10)	12 (24.00)	39 (34.21)		
Hypertension, *n* (%)				χ^2^ = 3.67	0.055
0	107 (65.24)	38 (76.00)	69 (60.53)		
1	57 (34.76)	12 (24.00)	45 (39.47)		
Diabetes, *n* (%)				χ^2^ = 0.00	0.949
0	134 (81.71)	41 (82.00)	93 (81.58)		
1	30 (18.29)	9 (18.00)	21 (18.42)		
Coronary heart disease, *n* (%)				χ^2^ = 0.40	0.528
0	149 (90.85)	47 (94.00)	102 (89.47)		
1	15 (9.15)	3 (6.00)	12 (10.53)		
Shock, *n* (%)				χ^2^ = 1.00	0.316
0	117 (71.34)	33 (66.00)	84 (73.68)		
1	47 (28.66)	17 (34.00)	30 (26.32)		
Prognosis, *n* (%)				χ^2^ = 0.29	0.587
1	97 (59.15)	28 (56.00)	69 (60.53)		
2	67 (40.85)	22 (44.00)	45 (39.47)		

t, *t*-test; Z, Mann-Whitney test; χ^2^, Chi-square test; SD, standard deviation; M, Median; Q1, 1st Quartile; Q3, 3rd Quartile; SIRS, Systemic Inflammatory Response Syndrome; GCS, Glasgow Coma Scale; PH, potential of hydrogen; PaO_2_, Arterial oxygen partial pressure; FiO_2_, Inhaled oxygen concentration; Lac, Lactate; PCT, procalcitonin; BNP, Brain natriuretic peptide; WBC, white blood cell; LY, Lymphocyte Count; HGB, hemoglobin; PLT, platelets; TBIL, Total Bilirubin; ALB, albumin; BUN, urea nitrogen; CREA, serum creatinine; CRP, C-reactive protein; GLU, blood glucose; PT, prothrombin time; APTT, ctivated partial thromboplastin time; INR, international normalized ratio; FIB, fibrinogen; DD, D-dimer; FDP, fibrinogen degradation products.

Within the training set, a comparative analysis between the survivor (*n* = 69) and non-survivor (*n* = 45) groups revealed statistically significant differences (*P* < 0.05) in several variables, as detailed in (Table 2). Specifically, the non-survivor group exhibited significantly elevated levels of lactate (2.70 vs. 1.50, *P* = 0.001), D-dimer (9.83 vs. 5.41, *P* = 0.005), and fibrinogen degradation products (21.57 vs. 13.32, *P* = 0.029). Furthermore, pulse rate was significantly higher in non-survivors (119.00 vs. 104.00, *P* = 0.011), while the length of ICU stay was significantly shorter (5.00 vs. 11.00 days, *P* < 0.001) (Table 2).

**Table 2:** Baseline characteristics and comparative analysis.

Variables	Total (*n* = 114)	No death (*n* = 69)	Death (*n* = 45)	Statistic	*P*
Age, Mean ± SD	63.10 ± 16.28	62.00 ± 16.76	64.78 ± 15.54	t = −0.89	0.376
APACHEII, M (Q1, Q3)	17.00 (13.00, 23.00)	17.00 (13.00, 23.00)	17.00 (13.00, 23.00)	Z = −0.12	0.901
SOFA, M (Q1, Q3)	6.00 (4.00, 8.00)	6.00 (4.00, 8.00)	6.00 (4.00, 9.00)	Z = −0.99	0.321
GCS, M (Q1, Q3)	15.00 (11.00, 15.00)	15.00 (11.00, 15.00)	15.00 (11.00, 15.00)	Z = −0.03	0.974
PH, M (Q1, Q3)	7.42 (7.34, 7.48)	7.45 (7.35, 7.48)	7.40 (7.33, 7.48)	Z = −1.09	0.274
Pa0_2_, M (Q1, Q3)	68.00 (55.92, 78.00)	71.30 (58.00, 79.00)	63.00 (55.00, 75.00)	Z = −1.67	0.095
FiO_2_, M (Q1, Q3)	0.60 (0.50, 0.80)	0.60 (0.45, 0.80)	0.60 (0.50, 1.00)	Z = −1.52	0.127
Lac, M (Q1, Q3)	1.85 (1.30, 3.10)	1.50 (1.20, 2.50)	2.70 (1.30, 5.60)	Z = −3.19	**0.001**
PCT, M (Q1, Q3)	1.12 (0.22, 5.71)	1.24 (0.25, 4.23)	0.99 (0.20, 8.98)	Z = −0.26	0.792
BNP, M (Q1, Q3)	385.10 (171.00, 858.21)	362.00 (158.00, 1,198.00)	387.00 (225.00, 712.87)	Z = −0.07	0.947
WBC, M (Q1, Q3)	10.92 (7.28, 15.13)	11.04 (7.17, 15.28)	10.80 (7.83, 14.90)	Z = −0.28	0.779
LY, M (Q1, Q3)	0.47 (0.30, 0.89)	0.52 (0.34, 0.92)	0.42 (0.25, 0.83)	Z = −1.15	0.252
HGB, M (Q1, Q3)	128.50 (112.00, 155.00)	131.00 (108.00, 158.00)	127.00 (119.00, 154.00)	Z = −0.14	0.889
PLT, M (Q1, Q3)	163.00 (111.50, 222.75)	163.00 (111.00, 207.00)	163.00 (113.00, 268.00)	Z = −0.71	0.476
TBIL, M (Q1, Q3)	20.09 (14.22, 27.67)	18.90 (13.00, 25.70)	23.20 (15.95, 30.90)	Z = −1.48	0.138
ALB, M (Q1, Q3)	27.45 (24.55, 30.58)	27.80 (24.70, 31.20)	27.20 (24.00, 29.10)	Z = −1.35	0.179
BUN, M (Q1, Q3)	7.75 (5.82, 11.68)	7.66 (4.84, 11.64)	8.20 (5.88, 11.84)	Z = −0.74	0.456
CREA, M (Q1, Q3)	71.00 (57.25, 100.75)	70.00 (58.00, 105.00)	74.45 (53.00, 97.00)	Z = −0.13	0.894
K, M (Q1, Q3)	3.87 (3.55, 4.23)	3.90 (3.52, 4.27)	3.86 (3.64, 4.17)	Z = −0.93	0.351
Na, M (Q1, Q3)	138.00 (134.00, 141.00)	138.00 (134.00, 140.00)	138.00 (134.00, 141.00)	Z = −0.76	0.445
Cl, M (Q1, Q3)	104.35 (99.35, 106.90)	104.30 (99.90, 106.90)	104.40 (98.90, 106.90)	Z = −0.54	0.592
Ca, M (Q1, Q3)	1.95 (1.84, 2.05)	1.94 (1.84, 2.05)	1.96 (1.84, 2.05)	Z = −0.89	0.372
P, M (Q1, Q3)	1.02 (0.77, 1.31)	1.00 (0.75, 1.30)	1.03 (0.81, 1.32)	Z = −0.60	0.550
Mg, M (Q1, Q3)	0.84 (0.75, 0.91)	0.85 (0.77, 0.90)	0.83 (0.75, 0.93)	Z = −0.07	0.945
CRP, M (Q1, Q3)	11.49 (6.45, 19.96)	10.65 (5.61, 19.08)	14.78 (9.35, 24.24)	Z = −1.85	0.064
GLU, M (Q1, Q3)	7.62 (6.04, 10.13)	7.59 (6.05, 10.19)	7.69 (5.96, 9.66)	Z = −0.02	0.981
PT, M (Q1, Q3)	14.25 (13.20, 16.38)	14.20 (13.20, 15.90)	14.30 (13.20, 16.90)	Z = −0.54	0.590
APTT, M (Q1, Q3)	33.20 (27.45, 43.15)	33.20 (27.90, 39.60)	32.50 (27.30, 45.70)	Z = −0.37	0.715
INR, M (Q1, Q3)	1.23 (1.11, 1.42)	1.24 (1.11, 1.38)	1.21 (1.11, 1.54)	Z = −0.04	0.965
FIB, M (Q1, Q3)	4.17 (3.18, 5.68)	3.84 (3.19, 5.27)	4.52 (3.18, 5.69)	Z = −0.57	0.568
DD, M (Q1, Q3)	5.86 (3.75, 12.26)	5.41 (3.13, 9.50)	9.83 (4.47, 16.47)	Z = −2.83	**0.005**
FDP, M (Q1, Q3)	15.43 (8.73, 27.87)	13.32 (8.32, 25.13)	21.57 (10.87, 39.75)	Z = −2.19	**0.029**
Body temperature, M (Q1, Q3)	36.80 (36.50, 37.30)	36.90 (36.50, 37.30)	36.70 (36.40, 37.20)	Z = −1.22	0.223
Pulse, M (Q1, Q3)	110.00 (97.00, 125.75)	104.00 (93.00, 118.00)	119.00 (104.00, 134.00)	Z = −2.53	**0.011**
Breathe, M (Q1, Q3)	28.00 (23.00, 35.00)	27.00 (22.00, 35.00)	28.00 (23.00, 36.00)	Z = −0.52	0.602
SBP, M (Q1, Q3)	129.00 (111.00, 148.75)	132.00 (112.00, 150.00)	124.00 (103.00, 141.00)	Z = −1.58	0.113
DBP, M (Q1, Q3)	79.00 (69.00, 88.00)	78.00 (65.00, 88.00)	80.00 (70.00, 88.00)	Z = −0.46	0.643
SIRS, M (Q1, Q3)	2.00 (2.00, 3.00)	2.00 (2.00, 3.00)	2.00 (2.00, 3.00)	Z = −0.48	0.633
Gender, *n* (%)				χ^2^ = 1.88	0.170
1	75 (65.79)	42 (60.87)	33 (73.33)		
2	39 (34.21)	27 (39.13)	12 (26.67)		
Hypertension, *n* (%)				χ^2^ = 0.24	0.628
0	69 (60.53)	43 (62.32)	26 (57.78)		
1	45 (39.47)	26 (37.68)	19 (42.22)		
Diabetes, *n* (%)				χ^2^ = 0.71	0.398
0	93 (81.58)	58 (84.06)	35 (77.78)		
1	21 (18.42)	11 (15.94)	10 (22.22)		
Coronary heart disease, *n* (%)				χ^2^ = 1.21	0.271
0	102 (89.47)	64 (92.75)	38 (84.44)		
1	12 (10.53)	5 (7.25)	7 (15.56)		
Shock, *n* (%)				χ^2^ = 1.89	0.169
0	84 (73.68)	54 (78.26)	30 (66.67)		
1	30 (26.32)	15 (21.74)	15 (33.33)		

t, *t*-test; Z, Mann-Whitney test; χ^2^, Chi-square test; SD, standard deviation; M, Median; Q1, 1st Quartile; Q3, 3st Quartile.

### 3.2 Feature selection and optimization

**Figure 1:**
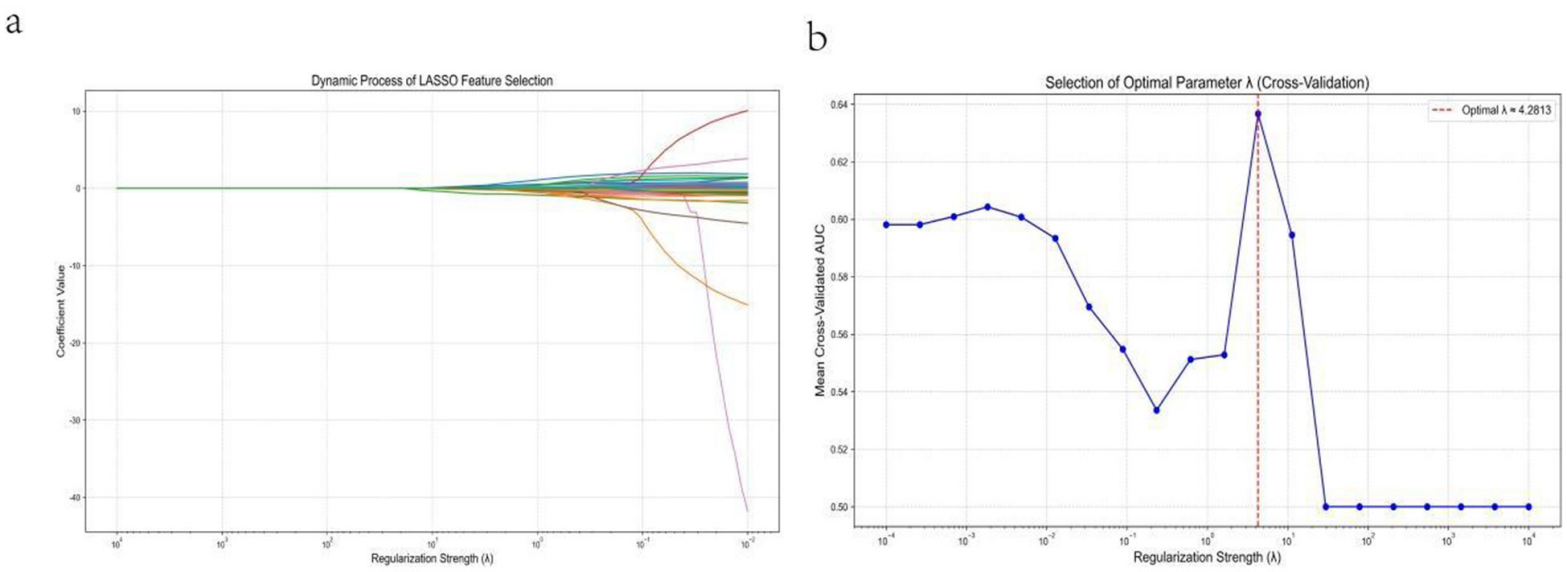
LASSO-based feature selection and optimization of the regularization parameter. Panel **(a)** shows the coefficient paths of candidate predictors as a function of the regularization strength (λ), illustrating progressive coefficient shrinkage toward zero and retention of variables with non-zero coefficients at the selected λ. Panel **(b)** shows 10-fold cross-validation results in the training set for selecting the optimal λ based on the mean cross-validated AUC, with the red dashed vertical line indicating the chosen value (λ ≈ 4.28).

Initially, Lasso regression Q10was employed to screen the initial 45 clinical variables. The feature coefficients exhibited contraction as the regularization strength increased. The optimal regularization parameter (λ) was determined through 10-fold cross-validation, ultimately selecting 17 features with non-zero coefficients. These features encompass key dimensions including physiological status, laboratory parameters, and clinical scores, specifically: FiO2 (fraction of inspired oxygen), Lac (lactate), DD (D-dimer), Temperature (body temperature), PT (prothrombin time), ALB (albumin), GLU (glucose), SBP (systolic blood pressure), GCS (Glasgow Coma Scale), WBC (white blood cell count), pH (blood pH), TBIL (total bilirubin), Ca (calcium), CREA (creatinine), Diabetes (presence of diabetes), APACHE II (APACHE II score), and Gender (Figure 1).

### 3.3 Model construction

Based on the selected features, we employed six machine learning algorithms to build predictive models, including: Gradient Boosting Decision Tree (GBDT), Random Forest (RF), Extreme Gradient Boosting (XGBoost), Support Vector Machine (SVM), Logistic Regression (LR), and Decision Tree (DT).

### 3.4 Model performance comparison and selection

**Figure 2:**
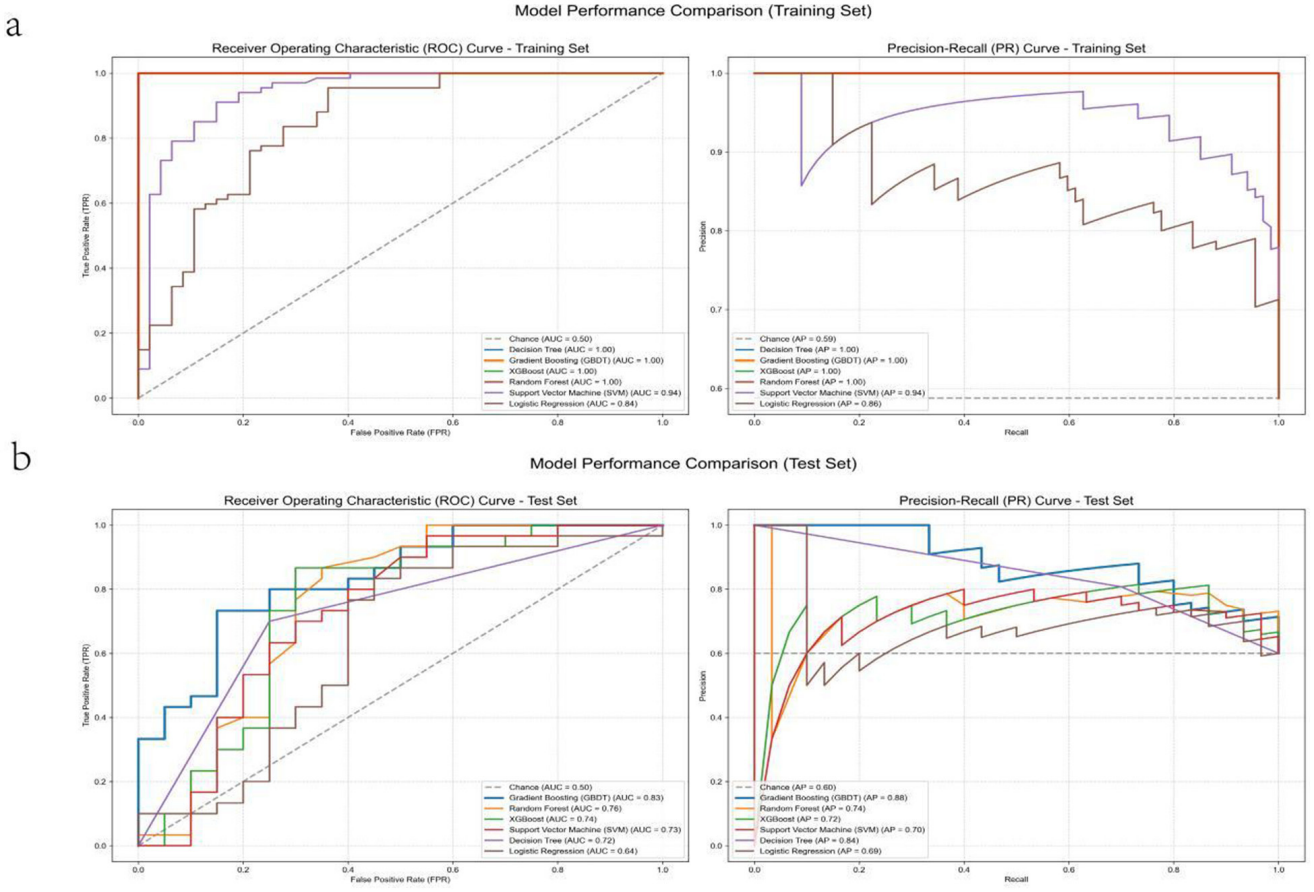
Performance comparison of machine-learning models on the training and test sets. **(a)** Training set and **(b)** test set: ROC curves **(left)** and precision–recall curves **(right)** for the evaluated classifiers; AUC and average precision (AP) are reported in the legends.

**Figure 3:**
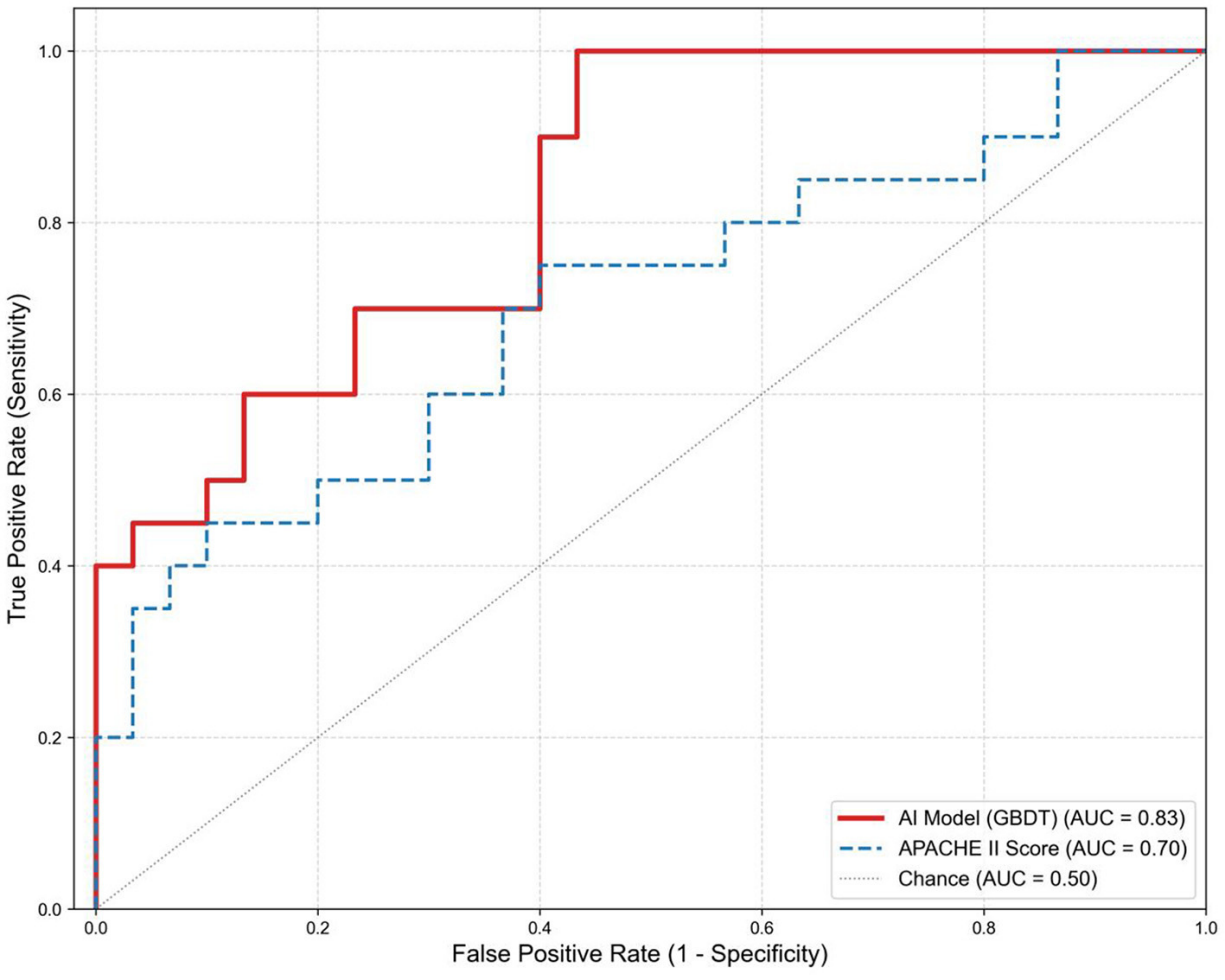
ROC comparison of the GBDT model and APACHE II score. The GBDT model (red) shows better discrimination than the APACHE II score (blue), with AUCs of 0.83 and 0.70, respectively; the gray diagonal indicates chance performance.

To evaluate the discriminative capability and positive-class recognition quality of different classification models, ROC and precision–recall (PR) curves were plotted for the training set and the validation set, with AUC and average precision (AP) as the primary metrics. In the training set, most tree-based/high-capacity models approached saturation: Decision Tree, Random Forest, GBDT, and XGBoost all achieved AUC = 1.00 and AP = 1.00, whereas SVM (AUC = 0.94, AP = 0.94) and Logistic Regression (AUC = 0.84, AP = 0.86) showed lower performance, suggesting limited capacity for linear or lower-complexity models. In the validation set, performance diverged more clearly: GBDT performed best (AUC = 0.83, AP = 0.88), followed by Random Forest (AUC = 0.76, AP = 0.74); XGBoost and SVM showed moderate performance (XGBoost: AUC = 0.74, AP = 0.72; SVM: AUC = 0.73, AP = 0.70), while Decision Tree achieved a relatively high AP (AP = 0.84) despite a lower AUC (AUC = 0.72), indicating less stable discrimination than ensemble methods; Logistic Regression performed worst (AUC = 0.64, AP = 0.69). Considering both training and validation/test results, GBDT demonstrated the best overall and more stable performance and was therefore selected as the final model for subsequent analyses and application (Figures 2 and 3).

### 3.5 Model calibration and clinical utility

**Figure 4:**
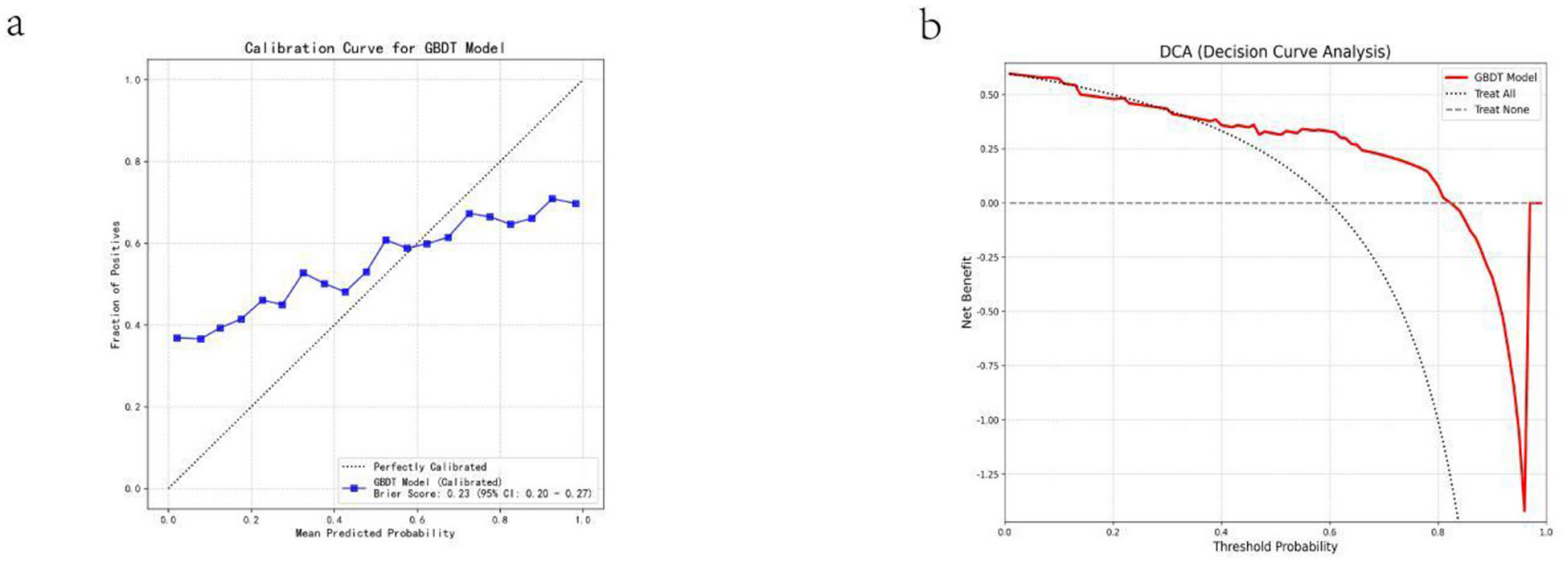
Calibration and decision-curve analysis of the GBDT model. **(a)** Calibration curve of the calibrated GBDT model (Brier score: 0.23; 95% CI: 0.20–0.27) against the ideal 45° line. **(b)** Decision curve analysis showing net benefit of the GBDT model vs. treat-all and treat-none across threshold probabilities.

The optimal GBDT model was calibrated and evaluated using 200 bootstrap resamples (Figure 4). The calibration curve closely approximated the ideal diagonal within the moderate prediction probability range, indicating acceptable agreement between predicted and observed risks. The model also achieved a Brier score of 0.23 (95% CI: 0.20–0.27), further supporting acceptable probabilistic accuracy on the validation set. Decision curve analysis (DCA) was performed to assess the clinical utility of using the model to trigger an early ICU enhanced high-risk management pathway (e.g., closer monitoring, more frequent reassessment, and early senior/multidisciplinary review) based on predicted mortality risk. The GBDT model demonstrated a consistently higher net benefit than “treat-all” and “treat-none” strategies across threshold probabilities of approximately 0.05–0.80, with clinically actionable gains particularly evident within the 0.10–0.60 range (Figure 4).

### 3.6 Model interpretability analysis

**Figure 5:**
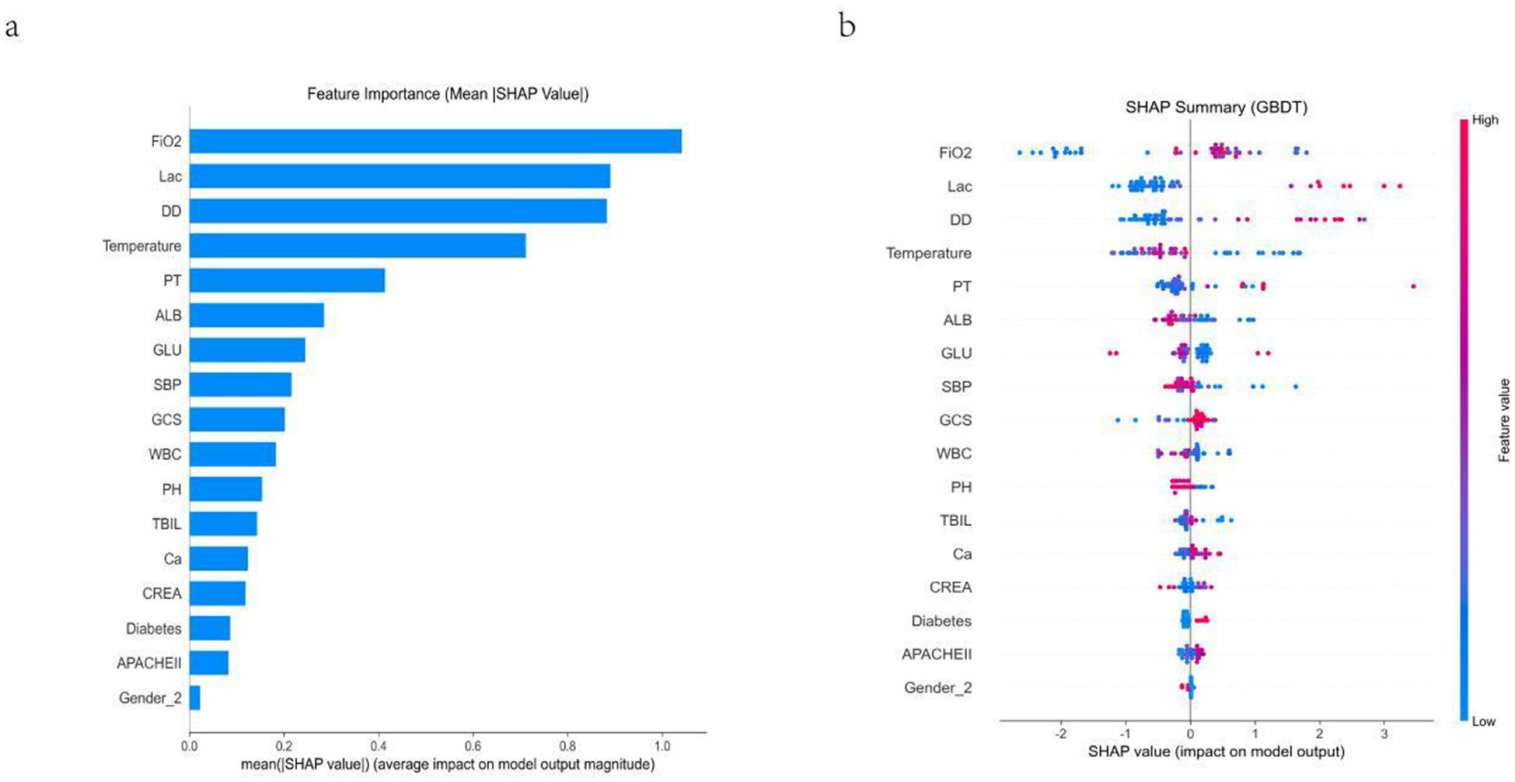
SHAP interpretation of the GBDT model. **(a)** Global feature importance ranked by mean absolute SHAP value. **(b)** SHAP summary (beeswarm) plot showing the direction and magnitude of each feature's contribution to the model output; color indicates feature value (low to high).

SHAP analysis results indicate that the features contributing most significantly to prediction outcomes in the GBDT model are, in descending order: Lactate (Lac), D-dimer (DD), Temperature, Albumin (ALB), Fraction of Inspired Oxygen (FiO_2_), and Prothrombin Time (PT). Overall, SHAP results reveal that model risk prediction is primarily driven by indicators related to coagulation function, lactate metabolism, and circulatory function, demonstrating good clinical interpretability (Figure 5).

### 3.7 Nomogram

**Figure 6:**
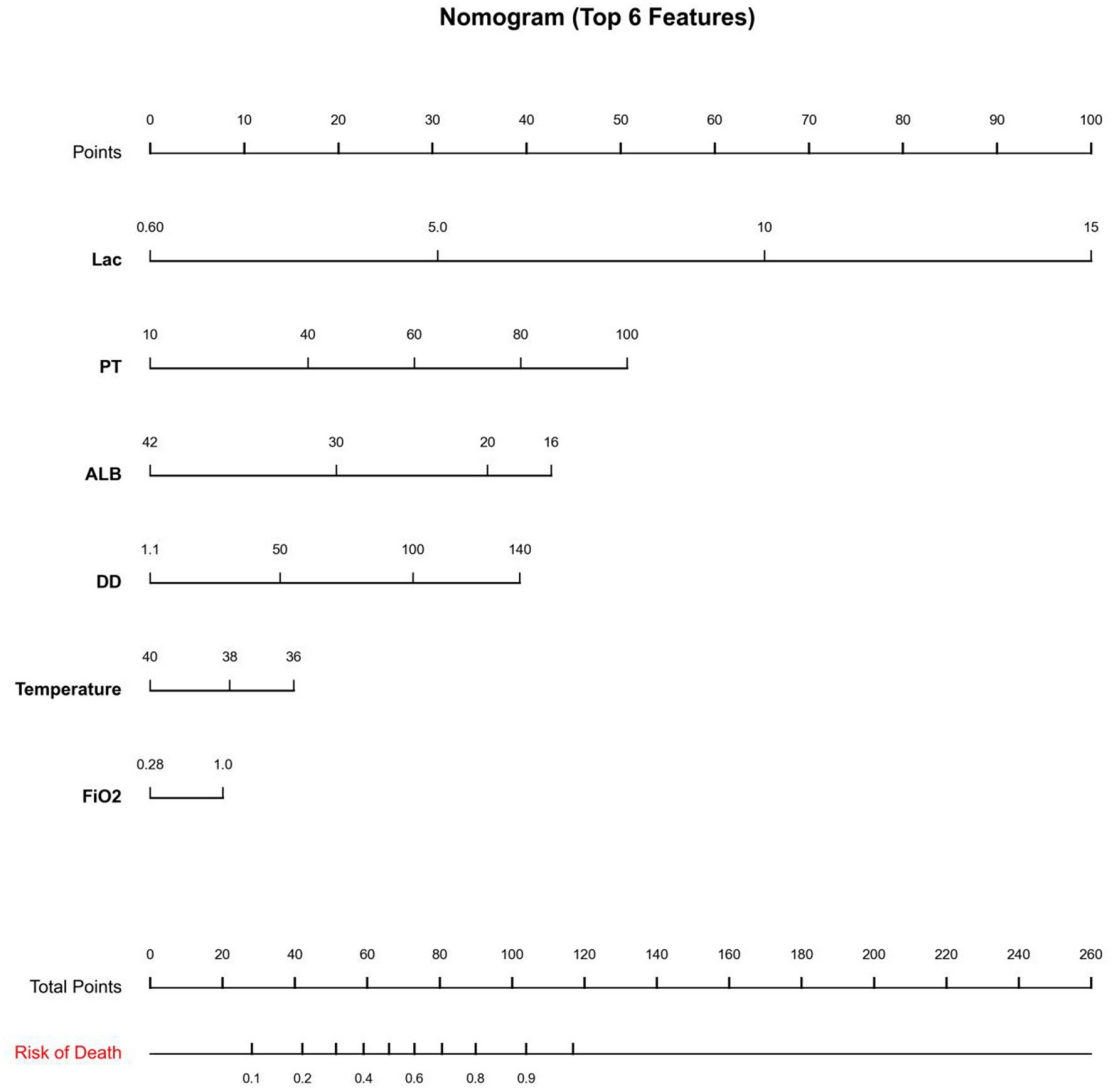
Nomogram for predicting mortality risk using the top six predictors. Points are assigned for Lac, PT, ALB, DD, temperature, and FiO_2_, summed as total points to estimate the probability of death.

To facilitate bedside implementation of the final model, we constructed a simplified nomogram using the most influential predictors identified by SHAP (Figure 6). The nomogram incorporated six key variables—lactate (Lac), Temperature, D-dimer (DD), albumin (ALB), Fraction of Inspired Oxygen (FiO_2_), and Prothrombin Time (PT). Each variable was assigned a point value according to its contribution, and the summed total points were mapped to an estimated probability of in-hospital mortality. This visualization provides an intuitive tool for individualized risk quantification using routinely available measurements and translates the model's core predictors into a clinically interpretable scoring system (Figure 6).

## 4 Discussion

This retrospective study developed and validated a machine-learning model to predict in-hospital mortality in ICU patients with severe community-acquired pneumonia complicated by respiratory failure. Among the candidate algorithms, the GBDT model showed the best generalization performance in the validation set (ROC-AUC = 0.83; AP = 0.88) and clearly outperformed the conventional APACHE-II score (AUC = 0.70), suggesting that a tailored model based on routinely available clinical variables can provide more accurate risk stratification than a generalized severity scoring system. More broadly, the growing use of machine learning for medical prediction and decision support across diverse diseases and data modalities—including optimization-enhanced neural networks, meta-ensemble frameworks, and transfer-learning–based approaches—highlights the feasibility and clinical relevance of ML-based modeling in healthcare (26–30). Within this context, our results support the value of a disease-specific, tabular-data model for ICU mortality risk stratification in SCAP with respiratory failure.

In addition to discrimination, we assessed probability reliability and potential clinical usefulness. The bootstrap-based calibration curve (200 resamples) demonstrated an overall close agreement between predicted and observed outcomes in the mid-probability range, supporting the reliability of the estimated mortality probabilities. Quantitatively, the model achieved a Brier score of 0.23 (95% CI: 0.20–0.27) on the validation set, further supporting acceptable probabilistic accuracy. Decision curve analysis further indicated that the GBDT model achieved a consistently higher net benefit than “treat-all” and “treat-none” strategies across a wide range of threshold probabilities (approximately 0.05–0.80), implying meaningful clinical utility for decision-making within commonly acceptable thresholds. In line with the intended use as an early ICU risk-stratification tool to trigger an enhanced high-risk management pathway (e.g., closer monitoring, more frequent reassessment, and early senior/multidisciplinary review), the clinically actionable threshold range is considered approximately 0.10–0.60, within which the model maintained clear net benefit. Consistently, the confusion matrix on the validation set showed high sensitivity (93.3%) with acceptable specificity (80.0%), indicating that the model is particularly effective in identifying patients at high risk of mortality.

To enhance interpretability, SHAP analysis was applied to the final GBDT model. The six leading contributors to model output were fraction of inspired oxygen (FiO2), lactate (Lac), D-dimer (DD), body temperature, prothrombin time (PT), and albumin (ALB). Elevated lactate showed a strong contribution toward higher predicted mortality, reflecting tissue hypoperfusion and metabolic stress in critical illness and severe infection (31, 32). Increased DD and PT were also associated with increased risk, highlighting inflammation-driven coagulation activation and hemostatic dysregulation in severe pneumonia and critical illness (33, 34). In contrast, higher albumin was predominantly protective, consistent with albumin as an integrated marker of inflammatory burden and nutritional/physiological reserve; hypoalbuminemia may indicate systemic inflammation, capillary leak, and reduced compensatory capacity (35, 36). Finally, greater oxygen requirements (higher FiO2) and abnormal thermoregulation were linked to higher mortality risk, consistent with more severe respiratory dysfunction and systemic derangement in critically ill patients (37). In addition, a nomogram was developed based on these six SHAP-identified predictors (FiO2, Lac, DD, body temperature, PT, and ALB) to facilitate bedside risk stratification and clinical implementation. By assigning points to each variable and summing them to obtain a total score, the nomogram provides an individualized estimate of mortality risk, enabling rapid and intuitive clinical decision support.

Several limitations should be acknowledged. First, the retrospective single-center design and the relatively small sample size may limit generalizability. Second, the near-saturated performance on the training set compared with the validation set suggests that residual overfitting cannot be fully excluded. Third, electronic medical record data may be subject to missingness and heterogeneity. In addition, net benefit estimates at very high threshold probabilities may be less stable in a small validation cohort due to sparse observations at extreme predicted risks. Future studies should include larger multicenter prospective cohorts for external validation, further calibration refinement (especially at probability extremes), and evaluation of real-world effectiveness when integrated into clinical workflows.

## 5 Conclusion

In conclusion, the proposed GBDT model demonstrated good discrimination (AUC = 0.83; AP = 0.88) and outperformed the conventional APACHE-II score for predicting in-hospital mortality in ICU patients with severe community-acquired pneumonia and respiratory failure. The model showed acceptable calibration and provided superior net benefit across a broad range of decision thresholds, supporting its potential clinical utility. SHAP interpretation identified Lac, DD, FiO2, ALB, Temperature, and PT as the key drivers of risk prediction, offering transparent and clinically meaningful insights for early identification of high-risk patients. Further multicenter prospective validation is warranted to confirm generalizability and facilitate implementation in routine practice.

## Data Availability

The original contributions presented in the study are included in the article/supplementary material, further inquiries can be directed to the corresponding author.
